# Genomic Epidemiology and Antimicrobial Susceptibility Profile of Enterotoxigenic *Escherichia coli* From Outpatients With Diarrhea in Shenzhen, China, 2015–2020

**DOI:** 10.3389/fmicb.2021.732068

**Published:** 2021-10-28

**Authors:** Chao Yang, Yinghui Li, Le Zuo, Min Jiang, Xianglilan Zhang, Li Xie, Miaomiao Luo, Yiying She, Lei Wang, Yixiang Jiang, Shuang Wu, Rui Cai, Xiaolu Shi, Yujun Cui, Chengsong Wan, Qinghua Hu

**Affiliations:** ^1^Shenzhen Center for Disease Control and Prevention, Shenzhen, China; ^2^Department of Microbiology, School of Public Health, Southern Medical University, Guangzhou, China; ^3^State Key Laboratory of Pathogen and Biosecurity, Beijing Institute of Microbiology and Epidemiology, Beijing, China; ^4^School of Public Health, University of South China, Hengyang, China; ^5^School of Public Health, Shanxi Medical University, Taiyuan, China

**Keywords:** enterotoxigenic *Escherichia coli* (ETEC), molecular epidemiology, whole genome sequencing (WGS), virulence factor, antimicrobial resistance (AMR), pathogenicity, public health surveillance

## Abstract

Enterotoxigenic *Escherichia coli* (ETEC) is the leading cause of severe diarrhea in children and the most common cause of diarrhea in travelers. However, most ETEC infections in Shenzhen, China were from indigenous adults. In this study, we characterized 106 ETEC isolates from indigenous outpatients with diarrhea (77% were adults aged >20 years) in Shenzhen between 2015 and 2020 by whole-genome sequencing and antimicrobial susceptibility testing. Shenzhen ETEC isolates showed a remarkable high diversity, which belonged to four *E. coli* phylogroups (A: 71%, B1: 13%, E: 10%, and D: 6%) and 15 ETEC lineages, with L11 (25%, O159:H34/O159:H43, ST218/ST3153), novel L2/4 (21%, O6:H16, ST48), and L4 (15%, O25:H16, ST1491) being major lineages. Heat-stable toxin (ST) was most prevalent (76%, STh: 60% STp: 16%), followed by heat-labile toxin (LT, 17%) and ST + LT (7%). One or multiple colonization factors (CFs) were identified in 68 (64%) isolates, with the common CFs being CS21 (48%) and CS6 (34%). Antimicrobial resistance mutation/gene profiles of genomes were concordant with the phenotype testing results of 52 representative isolates, which revealed high resistance rate to nalidixic acid (71%), ampicillin (69%), and ampicillin/sulbactam (46%), and demonstrated that the novel L2/4 was a multidrug-resistant lineage. This study provides novel insight into the genomic epidemiology and antimicrobial susceptibility profile of ETEC infections in indigenous adults for the first time, which further improves our understanding on ETEC epidemiology and has implications for the development of vaccine and future surveillance and prevention of ETEC infections.

## Introduction

Enterotoxigenic *Escherichia coli* (ETEC) is the leading cause of severe diarrhea particularly among young children aged less than five in developing countries, and is also the most common cause of diarrhea in travelers to ETEC-endemic areas, accounting for more than 200 million diarrheal cases and 50,000 deaths annually (Khalil et al., 2018; Fleckenstein and Kuhlmann, 2019). ETEC is defined by production of heat-stable toxin (ST) and/or heat-labile toxin (LT), and ST includes two subtypes, human ST (STh) and porcine ST (STp). STh is the most prevalent enterotoxin associated with human diarrhea, while STp is originally isolated from a porcine source and more prevalent in isolates from animals (So et al., 1976, 1978). There is a remarkable genetic diversity of ST and LT, and multiple variants have been identified (Joffré et al., 2015, 2016). In addition to enterotoxins, most ETEC isolates express one or more plasmid-encoded colonization factors (CF), which are fimbrial or afimbrial surface structures that enable adherence to intestinal epithelium. At least 27 known or putative CFs have been identified to date, including a novel CF, CS30 identified by genomic analysis, and the most prevalent CFs are colonization factor antigen I (CFA/I) and *coli* surface antigens 1–6 (CS1–CS6) (Gaastra and Svennerholm, 1996; Nada et al., 2011; von Mentzer et al., 2017; Vidal et al., 2019). Besides the classic virulence factors of enterotoxins and CFs, multiple non-classic virulence factors have been identified in recent years, including a cytoplasmic protein (LeoA), three adhesins (Tia, TibA, and EtpA), an extracytoplasmic protein (CexE), a hemolysin (ClyA), two mucinases (EatA and YghJ), three iron acquisition systems (Irp1, Irp2, and FyuA), and an enteroaggregative heat-stable toxin 1 (EAST1) (Turner et al., 2006; Pilonieta et al., 2007; Del Canto et al., 2011; Gonzales et al., 2013; Luo et al., 2014; Sjöling et al., 2015).

Enterotoxigenic *Escherichia coli* is genetically highly diverse, and more than 100 serotypes have been identified in clinical isolates (Wolf, 1997; Isidean et al., 2011). Multi-locus sequence type (MLST)-based studies showed that ETEC isolates can be found across five *E. coli* phylogroups including A, B1, B2, D, and E, with A and B1 being more prevalent (Turner et al., 2006; Sahl et al., 2011). Whole genome sequencing (WGS) of a large collection of representative global ETEC isolates further identified 21 robust lineages (L1–L21) characterized by distinct enterotoxin and CF profiles, which belonged to phylogroups A (12 lineages), B1 (7 lineages), C (1 lineage), and E (1 lineage) (von Mentzer et al., 2014; Denamur et al., 2021). Despite the diversity, a clear association between lineage and enterotoxin, CF, serotype, and plasmid content was identified. For example, close related lineages L1 and L2 encoded CS1 + CS3 and CS2 + CS3 (with/without CS21), respectively, but shared common enterotoxin profiles (STh + LT) and O antigen (O6) (von Mentzer et al., 2014). In addition, WGS has been used to characterize the epidemiology of ETEC infections in Bangladesh and Chile, and identified remarkable diversity of local circulating phylogroups and lineages. However, the dominant pathogenic phylogroups were distinct, with more Bangladeshi isolates belonging to phylogroup B1 while most Chilean isolates were from phylogroup A (Sahl et al., 2017; Rasko et al., 2019).

With the widespread use of antimicrobial agents, antimicrobial resistance (AMR) has emerged in ETEC isolates from both children and travelers with diarrhea (Qadri et al., 2005). AMR to commonly used agents such as nalidixic acid (NAL), ampicillin (AMP), tetracycline (TET), and sulfonamides has been frequently detected in ETEC isolates in Peru (Rivera et al., 2010), Bangladesh (Begum et al., 2016), South Korea (Oh et al., 2014), and China (Li et al., 2017), and the emergence of extended-spectrum β-lactamase (ESBL)-producing ETEC poses a new challenge to clinical treatment and public health (Margulieux et al., 2018; Guiral et al., 2019). Moreover, high-level and multidrug-resistant (MDR) had developed in ETEC isolates, which might be related to heavy clinical use of antimicrobial agents (Begum et al., 2016; Li et al., 2017). Due to the increased AMR in many areas over time, azithromycin and fluoroquinolones have been used as the first-line drugs for ETEC infections (Xiang et al., 2020). However, azithromycin-resistant ETEC also emerged in multiple countries (Abraham et al., 2014; Begum et al., 2016; Xiang et al., 2020), and is highly prevalent in Shanghai, China (Xiang et al., 2020), highlighting the necessity of ongoing surveillance of AMR, especially to first-line drugs.

While ETEC mainly causes diarrhea among children and travelers, most ETEC infections in Shenzhen, a populous developed city in southern China, were from indigenous adults, and the genomic epidemiology of Shenzhen ETEC isolates remains unclear. In this study, we sequenced the whole genomes of 106 ETEC isolates from indigenous outpatients with diarrhea in Shenzhen between 2015 and 2020, and compared them to a global collection of representative *E. coli* and ETEC genomes (von Mentzer et al., 2014; Horesh et al., 2021), to characterize the genomic diversity and virulence factors of ETEC in Shenzhen. Moreover, we integrated WGS-based *in silico* AMR mutation/gene detection and antimicrobial susceptibility testing to characterize the AMR profiles of Shenzhen ETEC isolates.

## Materials and Methods

### Strain Collection

ETEC strains were isolated from stool samples of outpatients with diarrhea in 16 sentinel hospitals in Shenzhen, China during routine foodborne disease surveillance between 2015 and 2020 as previously described (Li et al., 2017). Stool samples were enriched or inoculated on selective medium to isolate common foodborne pathogens, including *Salmonella*, *Shigella*, *Vibrio cholerae*, *Vibrio parahaemolyticus*, *Staphylococcus aureus*, *E. coli* O157:H7, ETEC, enteropathogenic *E. coli*, enteroinvasive *E. coli*, enterohemorrhagic *E. coli*, *Bacillus cereus*, group A *Streptococcus*, and *Listeria monocytogenes*. For ETEC, stool samples were further inoculated on CHROMagar ECC plates and incubated at 37°C overnight. Then, three colonies were randomly selected and inoculated on triple sugar iron agar for incubation and identification by screening ST and LT genes using a modified molecular beacon-based multiplex real-time PCR assay (Chen Q. et al., 2014). ETEC isolates were defined by either ST or LT gene was positive, and a total of 106 ETEC isolates were included in this study.

### Whole Genome Sequencing and Genome Dataset

Genomic DNA was extracted using the QIAamp DNA Mini Kit (QIAGEN, Hilden, Germany) according to manufacturer’s instructions. Pair-end libraries with a mean insert size of 350 bp were prepared for sequencing using Illumina NovaSeq 6000 platforms. The average read length is 150 bp, and ∼1.2 Gb clean data were generated for each isolate on average. Short-read sequencing data of Shenzhen isolates have been deposited in the NCBI Sequence Read Archive under the BioProject PRJNA739477, and the accession numbers were listed in Supplementary Table 1.

A total of 177 genomes were analyzed in this study, including 106 newly sequenced genomes of Shenzhen isolates and 71 publicly available representative genomes. These representative genomes were from the largest 50 *E. coli* lineages (Horesh et al., 2021) and 21 global ETEC lineages (von Mentzer et al., 2014), representing the phylogenetic diversity of *E. coli* and ETEC.

### Genome Assembly, Annotation, and Pan-Genome Analysis

We performed *de novo* assembling of genomes using SPAdes v3.14.1 (Bankevich et al., 2012) and obtained the assembled genomes of 106 ETEC isolates. The average number of contigs and size of assemblies were 123 (61–244, >500 bp) and 5.0 Mb (4.7–5.2), with an average of 102-fold (88–128) depth for each genome.

The assembled genomes were annotated using Prokka (Seemann, 2014) with default settings, and the gene annotation results (GFF3 files) were used in Panaroo (Tonkin-Hill et al., 2020) to identify the pan-genome and generate the matrix of accessory gene presence/absence. Genes presented in ≥99% isolates were defined as core genes, and the other genes were defined as accessory genes. EggNOG-mapper (Huerta-Cepas et al., 2019) was used to annotate the Clusters of Orthologous Groups (COGs) classifications of accessory genes.

### Serotyping and Multi-Locus Sequence Typing

*In silico* serotyping was performed using ECTyper^1^ based on assembled genomes. MLST sequence type (MLST-ST) was obtained by scanning the sequences of seven house-keeping genes (*adk*, *fumC*, *gyrB*, *icd*, *mdh*, *purA*, and *recA*) against PubMLST typing schemes using mlst.^2^

### Virulence Factors and Plasmid Detection and Typing

The presence or absence of classic virulence genes (enterotoxins and CFs) and non-classic virulence genes were identified using ABRicate^3^ and BLASTN by scanning against the ETEC virulence database^4^ and a custom non-classic virulence genes database. We analyzed a total of 11 non-classic virulence genes, including *leoA* (accession number: AF170971), *tia* (U20318), *tibA* (AF109215), *etpA* (AY920525, position: 2718-8021), *cexE* (LR883053, position: 25424–25792), *clyA* (AY576657, position: 241–1152), *eatA* (AY163491), *yghJ* (DQ866820, position: 322–622), *irp2* (NC_003143, position: 2156049–2162156), *fyuA* (NC_003143.1, position: 2140840–2142861), and *astA* (AB042005). A gene was considered present if the overall coverage and identity were larger than 80%.

The ST and LT nucleotide sequences of Shenzhen isolates were extracted from assembled genomes using BLASTN and were aligned against the sequences of previously reported ST and LT variants^5^ (Joffré et al., 2015, 2016; von Mentzer et al., 2021) using MAFFT v7.480 (Katoh and Standley, 2013). Multiple sequence alignments were used to construct the maximum likelihood phylogenetic trees, and the ST and LT variants of Shenzhen isolates were determined based on the phylogenetic trees (Supplementary Figure 1).

PlasmidFinder v2.1 (Carattoli et al., 2014) was used to detect and determine the incompatibility groups of plasmids based on assembled genomes with default settings.

### Single-Nucleotide Polymorphism Calling and Phylogenetic Analyses

Core-genome (regions present in >99% strains) single-nucleotide polymorphisms (SNPs) were identified using Snippy v4.6.0 pipeline,^6^ with *E. coli* K12 (accession number: NC_000913) as the reference genome. Briefly, for each isolate, clean sequencing reads were mapped to reference genome using BWA-mem (Li and Durbin, 2009), and SNPs were called using SAMtools (Li et al., 2009) and FreeBayes (Garrison and Marth, 2012). Repetitive regions of the reference genome were identified using Tandem Repeats Finder (TRF) (Benson, 1999) and by self-aligning using BLASTN as previously described (Yang et al., 2019). SNPs located in repetitive regions were removed prior to phylogenetic analysis. The maximum likelihood trees based on non-repetitive core-genome SNPs and alignments of ST and LT nucleotide sequences were constructed using IQ-TREE v2.0.3 (Minh et al., 2020) with auto-detected best-fitting substitution model, respectively.

### Antimicrobial Resistance Mutation/Gene Detection and Phenotype Testing

ResFinder 4.1 (Bortolaia et al., 2020) was used to detect the AMR related mutations and genes based on clean sequencing reads with default settings. Based on the lineage classification and AMR mutation/gene distribution, we selected 52 representative isolates for AMR phenotype testing. The susceptibilities of the representative isolates were determined using disc diffusion assay against 17 antimicrobial agents, including NAL, AMP, TET, ampicillin/sulbactam (AMS), cefotaxime (CTX), trimethoprim/sulfamethoxazole (SXT), azithromycin (AZI), streptomycin (STR), ciprofloxacin (CIP), ceftazidime (CAZ), amikacin (AMK), chloramphenicol (CHL), colistin (CT), ceftazidime/avibactam (CZA), ertapenem (ETP), meropenem (MEM), and tigecycline (TGC). Breakpoints for sensitive, intermediate, and resistant were defined by the Clinical and Laboratory Standards Institute (CLSI) document M100-S26, and *E. coli* ATCC25922 was used as the control. ESBL-producing isolates were identified by assessing susceptibility phenotype to CTX, CAZ, and CZA.

## Results

### Demographic and Clinical Symptom Characteristics

A total of 106 ETEC isolates from indigenous outpatients with diarrhea collected during routine foodborne disease surveillance between 2015 and 2020 were included in this study (Supplementary Table 1). These outpatients had a median age of 27 years (interquartile range: 21–36), of which only seven (7%) was children aged <5 years and 82 (77%) were adults aged >20 years. There were more males (51%, 54/106) than females (49%, 52/106), and the clinical symptoms included diarrhea (95%, 100/106), abdominal pain (29%, 31/106), vomiting (8%, 9/106), and fever (8%, 9/106).

### *Escherichia coli* Phylogroup and Enterotoxigenic *Escherichia coli* Lineage

**FIGURE 1:**
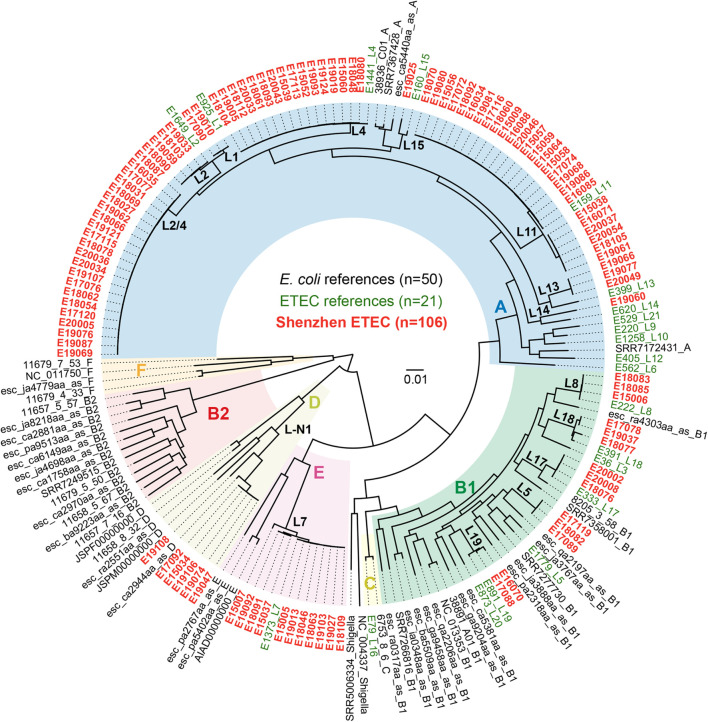
Maximum-likelihood phylogenetic tree of 177 *E. coli* and ETEC isolates. Colors of the tree tip labels indicated the source of isolates, black for representative *E. coli* isolates from the largest 50 *E. coli* lineages, green for representative isolates from 21 global ETEC lineages, and red for 106 Shenzhen ETEC isolates. Background colors of the phylogenetic branches indicated the *E. coli* phylogroups.

To investigate the *E. coli* phylogroup and ETEC lineage classification of Shenzhen isolates, we compared the 106 Shenzhen isolates to 71 representative isolates from the largest 50 *E. coli* lineages (Horesh et al., 2021) and 21 global ETEC lineages (von Mentzer et al., 2014), and constructed a maximum-likelihood (ML) tree of 177 isolates based on 267,822 SNPs in the core-genome (Figure 1). Shenzhen isolates showed a remarkably high diversity, which can be attributed to four *E. coli* phylogroups and 15 ETEC lineages (Table 1 and Figure 1). The majority of Shenzhen isolates belonged to phylogroup A (71%, 75/106), and the remaining were assigned into phylogroup B1 (13%, 14/106), D (6%, 6/106), and E (10%, 11/106). Phylogroup A can be further divided into eight ETEC lineages, and lineage L11 (25%, 26/106), a novel lineage L2/4 (between L2 and L4, 21%, 22/106, Figure 1), and L4 (15%, 16/106) were major circulating lineages. Phylogroup B1 can be divided into five lineages (L5, L8, and L17–L19) each with few isolates (*n* ≤ 3), and D and E each contained one lineage, a novel lineage L-N1 (*n* = 6) and L7 (*n* = 11), respectively (Figure 1).

**TABLE 1:** Classification, enterotoxin, and colonization factor distribution of 106 ETEC isolates in Shenzhen, 2015-2020.

*E. coli* phylogroup	ETEC lineage	No. of isolates	Serotype^1^	Sequence type (ST)^1^	Enterotoxin variant	Colonization factor	Non-classic virulence genes	Plasmid incompatibility groups
A (*n* = 75)	L1	2	O6:H16	ST6955	STh (STa3/4) + LT (LT20)	CS1 + CS3 + CS21	*tia*, *etpA*, *cexE*, *clyA*, *eatA*, *yghJ*, *astA*	FII, FIB, I1 (*n* = 1); FII, FIB, I1, B/O/K/Z (*n* = 1)
	L2	4	O6:H16	ST4	STh (STa3/4) + LT (LT20)	CS2 + CS3 + CS21	*etpA*, *clyA*, *eatA*, *yghJ*, *astA*	FII, FIB (*n* = 2); FII, FIB, I2 (*n* = 3)
	L2/4	22	O6:H16	ST48	STh (STa3/4, *n* = 21); STp (STa1, *n* = 1)	CS21 (*n* = 21)	*etpA*, *clyA*, *yghJ*, *astA* (*n* = 21); *etpA*, *clyA*, *eatA*, *yghJ*, *astA* (*n* = 1)	FII, FIB, I1 (*n* = 18); FII, FIB (*n* = 1); FII, FIB, I1, B/O/K/Z (*n* = 1); FII, I1, N (*n* = 1)
	L4	16	O25:H16	ST1491	LT (LT17)	CS6 + CS21	*clyA*, *eatA*, *yghJ*	FII, FIB (*n* = 4); FII, FIB, B/O/K/Z (*n* = 6); FII, FIB, B/O/K/Z, Y (*n* = 1); FII, FIB, I1 (*n* = 1)
	L11	26	O159:H34 (*n* = 20); O159:H43 (*n* = 6)	ST218 (*n* = 20), ST3153 (*n* = 6)	STh (STa3/4)		*etpA*, *clyA*, *yghJ*, *astA*	FII (*n* = 13); FII, B/O/K/Z (*n* = 7); FII, I1 (*n* = 6)
	L13	3	O-:H10	ST226	STh (STa3/4)		*etpA*, *clyA*, *yghJ*, *astA*	FII, I1
	L14	1	O25:H42	ST1201	STh (STa2)	CS4 + CS6	*clyA*, *eatA*, *yghJ*, *astA*	FII, FIB, B/O/K/Z
	L15	1	O107/117:H27	ST10	STh (STa7)	CS21	*etpA*, *clyA*, *eatA*, *yghJ*	FII, FIB
B1 (*n* = 14)	L5	3	O159:H20	ST2040	STh (STa3/4)		*etpA*, *clyA*, *yghJ*, *astA*	FII, B/O/K/Z
	L8	3	O148:H28	ST94	STh (STa7)	CS6 + CS21	*clyA*, *astA*	FII, FIB
	L17	3	O27:H7	ST316	STp (STa5)	CS6	*clyA*, *yghJ*	FII, FIB
	L18	3	O159:H34 (*n* = 2); O23:H16 (*n* = 1)	ST1490 (*n* = 2), ST- (*n* = 1)	STp (*n* = 2, STa5), LT (*n* = 1, LT29)	CS6 + CS23 (*n* = 2)	*clyA*, *eatA*, *yghJ*, *astA*	FII, FIB
	L19	2	O29:H12 (*n* = 1); O153:H12 (*n* = 1)	ST155	STh (*n* = 1, STa3/4), LT (*n* = 1, LT15)		*etpA*, *clyA*, *yghJ*, *astA* (*n* = 1); *clyA*, *yghJ* (*n* = 1)	FII (*n* = 1); FII, FIB (*n* = 1)
D (*n* = 6)	L-N1	6	O15:H18 (*n* = 5); O-/H18 (*n* = 1)	ST69 (*n* = 5), ST1380 (*n* = 1)	STh (*n* = 5, STa7), STp + LT (STa8 + LT4, *n* = 1)	CS21 (*n* = 4)	*etpA*, *clyA*, *eatA*, *yghJ*, *irp2*, *fyuA* (*n* = 5); *clyA*, *yghJ*, *astA* (*n* = 1)	FII, FIB (*n* = 4); FII (*n* = 1); FII, FIB, B/O/K/Z, FIA (*n* = 1
E (*n* = 11)	L7	11	O169:H41 (*n* = 10); O167:H41 (*n* = 1)	ST182 (*n* = 10), ST3930 (*n* = 1)	STp (STa5)	CS6	*leoA*, *tia*, *clyA*, *yghJ*, *astA* (*n* = 7); *clyA*, *yghJ*, *astA* (*n* = 4)	FII, FIB

*^1^Untypeable serotype O and MLST sequence type was denoted by O- and ST-, respectively.*

**FIGURE 2:**
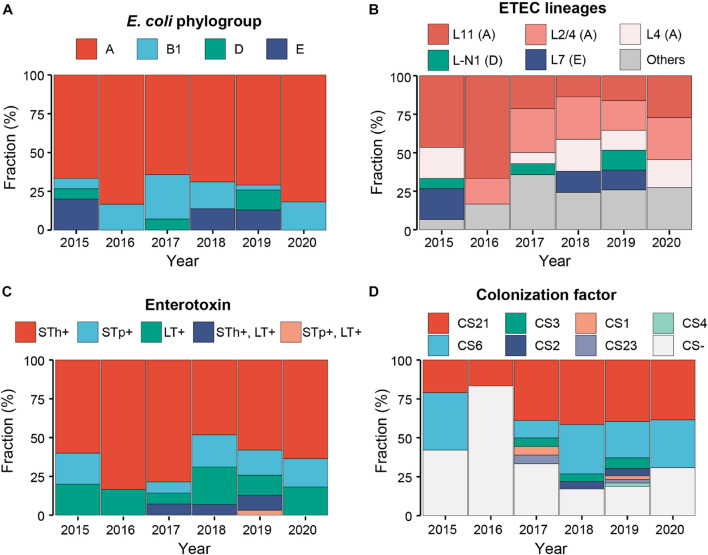
Temporal dynamics of the *E. coli* phylogroups **(A)**, ETEC lineages **(B)**, enterotoxins **(C)**, and colonization factors **(D)** of Shenzhen isolates during the 6-year sampling period. Colors indicated different classifications as shown in the legends above.

Phylogroup A has always been dominant during the 6-year sampling period, while the fractions of other phylogroups were variable. At the end of sampling in 2020, only two phylogroups A and B1 persisted (Figure 2A). The fractions of different ETEC lineages were also variable. Lineage L11 was dominant before 2016; since 2017, multiple lineages coexisted and no obvious dominant lineage was identified (Figure 2B).

### Serotype and Sequence Type

To compare with traditional subtyping results, we performed *in silico* serotyping and MLST-ST based on assembled genome sequences. There was a close association of ETEC lineage with serotype and MLST-ST, with isolates within a lineage belonging to 1–2 serotypes or STs (Table 1). The most prevalent serotype was O6:H16 (26%, 28/106), followed by O159:H34 (21%, 22/106) and O25:H16 (15%, 16/106). The most prevalent MLST-ST was ST48 (21%, 22/106), followed by ST218 (19%, 20/106) and ST1491 (15%, 16/106). The serotype and MLST-ST of three major lineages were O159:H34/O159:H43 and ST218/ST3153 for L11, O6:H16 and ST48 for L2/4, and O25:H16 and ST1491 for L4 (Table 1).

### Virulence Factors

Heat-stable toxin (ST)-positive ETEC has been most prevalent (76%, 81/106) in Shenzhen throughout the sampling period (Figure 2C), followed by LT-positive ETEC (17%, 18/106). ST and LT-positive ETEC was only detected in seven isolates (7%) in three of the 6-year sampling period. Among ST-positive ETEC, STh-positive isolates (60%, 64/106) was more prevalent than STp-positive ones (16%, 17/106) throughout the sampling period (Figure 2C). More specifically, the most prevalent ST variant was STa3/4 (57%, 60/106), followed by STa5 (15%, 16/106) and STa7 (8%, 9/106); the most prevalent LT variant was LT17 (15%, 16/106). Two novel variants of STp and LT, designated as STa8 and LT29, were identified in one isolate, respectively (Table 1, Supplementary Table 1, and Supplementary Figure 1).

One to three known CFs were identified in 68 (64%) Shenzhen isolates. The most prevalent CF was CS21 (48%, 51/106, with or without other CFs), followed by CS6 (34%, 36/106) throughout the sampling period (Figure 2D). Except CS21 and CS6, the fraction of other CFs was low and they were only detected in 3 years. The most prevalent combination was CS6 + CS21 (18%, 19/106).

In addition to enterotoxins and CFs, 10 of 11 detected non-classic virulence genes were identified in Shenzhen isolates with different frequencies (2–100%, Table 1 and Supplementary Table 1). The hemolysin gene *clyA* (100%, 106/106) and mucinase gene *yghJ* (97%, 103/106) were most prevalent in Shenzhen isolates, followed by enteroaggregative heat-stable toxin gene *astA* (75%, 79/106), adhesion gene *etpA* (63%, 67/106), and another mucinase gene *eatA* (30%, 32/106). The other five non-classic virulence genes were detected with low frequencies (2–8%). Co-presence of classic and non-classic virulence genes was identified. The *etpA* (94%, 60/64) and *astA* (91%, 58/64) genes were mostly detected along with STh, and *eatA* (89%, 16/18) gene was mostly detected along with LT.

The distribution of classic and non-classic virulence genes in different lineages was shown in Table 1 and Supplementary Table 1. Twelve (80%) of 15 lineages were ST-positive (53%, 8/15) or contained ST-positive isolates (27%, 4/15), and STh-positive lineages (40%, 6/15) or lineages containing STh-positive isolates (20%, 3/15) were more prevalent than STp-positive lineages (13%, 2/15) or lineages containing STp-positive isolates (13%, 2/15). CFs were detected in 11 lineages (73%), and the most prevalent CFs, CS21, and CS6 were detected in seven (47%) and six (40%) lineages, respectively. Except two prevalent non-classic virulence genes *clyA* and *yghJ*, *astA*, *etpA*, and *eatA* genes were detected in 12 (80%), 9 (60%), and 8 (53%) lineages, respectively.

In addition to the association between lineage and serotype and MLST-ST, the association of lineage with enterotoxin and CF was also identified. Most isolates within a lineage shared identical virulence gene (classic and non-classic) profiles, except for five lineages (L2/4, L7, L18, L19, and L-N1) in which two or three types of virulence gene combinations were detected. In contrast, the virulence gene profiles of different lineages were largely distinct (Table 1 and Supplementary Table 1).

### Plasmid Incompatibility Groups

*In silico* plasmid typing analysis identified replicons belonging to eight incompatibility (Inc) groups in all the 106 Shenzhen isolates, with one to four Inc groups present in each isolate (Table 1 and Supplementary Table 1). The replicon IncFII was most prevalent and can be detected in all Shenzhen isolates, followed by IncFIB (67%, 71/106), IncI1 (31%, 33/106), and IncB/O/K/Z (20%, 21/106); the other replicons (IncFIA, IncI2, LncN, and LncY) were only detected in one isolate, respectively. There was an association between the replication IncFIB and virulence gene CS21, and 72% (51/71) IncFII-positive isolates also encode CS21. Only one type of replicon profile was identified in eight lineages, and the other seven lineages each contained two to four types of replicon profiles (Table 1 and Supplementary Table 1).

### Pan-Genome Analysis

We performed pan-genome analysis of 127 ETEC genomes of Shenzhen isolates (*n* = 106) and globally representative isolates (*n* = 21) to identify genes unique to or significantly associated with Shenzhen isolates. A total of 11,813 pan-genes were identified in these 127 genomes, including 3,307 core-genes (present in ≥99% isolates) and 8,506 accessory genes. There were no genes unique to Shenzhen or non-Shenzhen isolates; however, we identified significant differences (*p* < 0.01, Fisher’s exact tests) in the frequencies of 589 accessory genes between Shenzhen and non-Shenzhen isolates (Supplementary Table 2). There were 178 accessory genes significantly enriched in Shenzhen isolates, of which two were known virulence genes STh and *etpA*, and the other genes encoded hypothetical proteins with unknown functions (46%) or were associated with replication and repair (COG category L; 37%) and cell wall/membrane/envelop biogenesis (COG category M; 19%). For example, STh was present in 66% Shenzhen isolates and 29% non-Shenzhen isolates, respectively; the gene *group_4545* encoding a hypothetical protein was present in 91% Shenzhen isolates but in only 19% non-Shenzhen isolates. In addition, there were 411 accessory genes significantly enriched in non-Shenzhen isolates including the virulence gene LT (Supplementary Table 2).

We further attempted to identify genes unique to two novel lineages L2/4 and L-N1. A total of 25 L2/4 lineage unique genes were identified (Supplementary Table 3), of which 76% encoded hypothetical proteins and 20% were associated with replication and repair (COG category L). There were 105 genes unique to L-N1 lineage (Supplementary Table 3), of which 72% encoded hypothetical proteins, 10% were associated with cell motility (COG category N) and intracellular trafficking and secretion (COG category U), and 5% were associated with transcription (COG category K). None of these L2/4 and L-N1 lineage unique genes were known ETEC virulence genes.

### Antimicrobial Susceptibility

**FIGURE 3:**
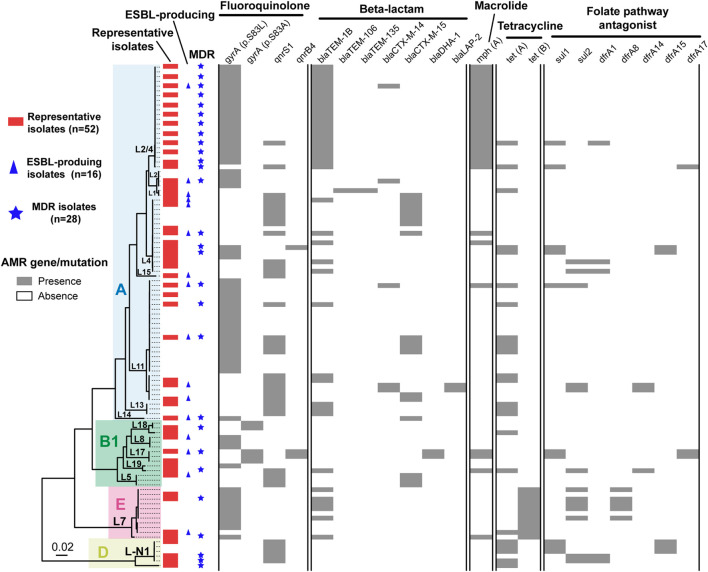
Antimicrobial resistance related mutation and gene distribution in Shenzhen isolates. Maximum likelihood tree of 106 Shenzhen isolates was shown on the left, and the background colors indicated the *E. coli* phylogroups as in Figure 1. The distribution of representative isolates for phenotype testing (red bars), extended-spectrum β-lactamase (ESBL)-producing isolates (blue triangles), multidrug-resistant (MDR) isolates (blue stars), and the presence (gray bars) or absence (white bars) of antimicrobial resistance related mutation and gene were shown on the right.

To characterize the AMR profiles of Shenzhen isolates, we firstly scanned the genomes to identify AMR related mutations and genes. A total of 21 AMR mutations/genes were detected (Figure 3), which were involved in resistance to five classes of antimicrobial agents, including fluoroquinolone (two mutations and two genes), beta-lactam (seven genes), tetracycline (two genes), macrolide (one gene), and folate pathway antagonist (seven genes).

Fluoroquinolone resistance mutations/genes were most prevalent, which were detected in 99 (93%) isolates. For example, DNA gyrase gene *gyrA* p.S83L mutation and quinolone resistance gene *qnrS1* were detected in 63 (59%) and 37 (35%) isolates, respectively. Beta-lactam resistance genes ranked second and were detected in 63 (59%) isolates, of which *bla*_*TEM*–1*B*_ and *bla*_*CTX*–*M*–15_ were more prevalent and were detected in 40 (38%) and 18 (17%) isolates, respectively. Tetracycline resistance genes were detected in 34 (32%) isolates, of which *tet(A)* and *tet(B)* were detected in 24 (23%) and 11 (10%) isolates, respectively. A macrolide resistance gene *mph(A)* was detected in 29 (27%) isolates. Notably, this gene has been reported to mediate azithromycin resistance, which is usually less frequent in ETEC (Xiang et al., 2020). Folate pathway antagonist resistance genes, including *sul1*, *sul2*, *dfrA1*, *dfrA8*, *dfrA14*, *dfrA15*, and *dfrA17* were detected in 22 (21%) isolates, and each gene appeared in 3–13 isolates.

Unlike the close association of lineage with serotype, MLST-ST and virulence factors, there was no obvious association between lineages and most AMR mutations/genes, with multiple AMR mutations/genes combinations being identified in isolates of a lineage (Figure 3). Notably, most isolates of two major lineages L11 and L2/4 had *gyrA* p.S83L mutations, and all the L2/4 lineage isolates also carried *bla*_*TEM*–1*B*_ and *mph(A)* genes.

We further selected 52 isolates representing all the lineages and AMR mutations/genes combinations for AMR phenotype testing against 17 antimicrobial agents (Table 2 and Figure 3). AMR mutations/genes distributions were generally concordant with phenotype testing results. The resistance to a quinolone agent, NAL was highest (71%, 37/52) in Shenzhen isolates, and 78% (29/37) of them carried *gyrA* (p.S83L or p.S83A) mutations. However, only two (4%) isolates were resistant to another quinolone agent ciprofloxacin. The resistance to beta-lactam agents, including AMP (69%, 36/52), AMS (46%, 24/52), and CTX (31%, 16/52) was also prevalent. Sixteen (31%) representative isolates were identified as ESBL-producing, of which 81% (13/16) encode at least one type of β-lactamase associated gene, with *bla*_*CTX*–*M*–15_ (50%, 8/16) and *bla*_*CTX*–*M*–14_ (25%, 4/16) being most common. In addition, the resistance to other three classes of antimicrobial agents were detected, including AZI (38%, 20/52, macrolide class), TET (37%, 19/52, tetracycline class), and SXT (29%, 15/52, folate pathway antagonist class). There was a close association between azithromycin resistance and the presence of *mph(A)* gene, with 18 of 20 azithromycin-resistant isolates encoding *mph(A)*. No resistance to seven antimicrobial agents was detected, including AMK, CHL, CT, CZA, ETP, MEM, and TGC. All (12/12) the tested representative L2/4 lineage isolates were resistant to at least three classes of agents, i.e., multidrug-resistant (MDR); 16 (40%) of 40 representative isolates of non-L2/4 lineages were MDR (Table 2 and Figure 3).

**TABLE 2:** Antimicrobial resistance of 52 representative Shenzhen ETEC isolates.

Antimicrobial agent^1^	Antimicrobial resistance rate (%)						
	L11 (*n* = 7)	L2/4 (*n* = 12)	L4 (*n* = 10)	L7 (*n* = 4)	L-N1 (*n* = 4)	Other^2^ (*n* = 15)	Total (*n* = 52)
NAL	71	92	60	75	50	67	71
AMP	86	100	40	75	25	67	69
AMS	29	100	20	25	0	47	46
AZI	14	100	20	25	0	27	38
TET	43	50	20	100	100	27	37
CTX	57	25	30	25	0	47	31
SXT	14	50	30	25	75	33	29
STR	43	25	0	25	50	20	19
CIP	0	0	10	0	0	7	4
CAZ	0	0	0	0	0	7	2
ESBL	57	8	30	25	0	47	31
MDR	43	100	30	50	75	33	54

*^1^Only 10 of 17 antimicrobial agents with at least one resistant isolate were shown. ^2^Lineages with <3 isolates together were defined as others. NAL, nalidixic acid; AMP, ampicillin; TET, tetracycline; AMS, ampicillin/sulbactam; CTX, cefotaxime; SXT, trimethoprim/sulfamethoxazole; AZI, azithromycin; STR, streptomycin; CIP, ciprofloxacin; CAZ, ceftazidime. ESBL, extended-spectrum β-lactamase; MDR, multidrug-resistant, defined as resistant to ≥3 classes of antimicrobial agents.*

## Discussion

Despite the high prevalence in children and travelers, ETEC also lead to substantial infections in adults in endemic areas (Lamberti et al., 2014). Moreover, it is intriguing that most ETEC infections in Shenzhen (Li et al., 2017), as well as several other developed cities and province in China (Chen Y. et al., 2014; Qu et al., 2014; Pan et al., 2015), such as Beijing and Shanghai, were from indigenous adults. However, there is currently a lack of understanding on the epidemiology of ETEC infections in indigenous adults. Compared to traditional molecular subtyping methods, WGS not only provides the ultimate resolution on reconstruction the relationships between isolates, but also can be used for secondary in-depth virulence factors and AMR genes analysis, which has been one of the most powerful methods to characterize the epidemiology of pathogens (Didelot et al., 2012; Allard et al., 2016; Ronholm et al., 2016). We reconstructed the WGS-based population structure of Shenzhen isolates in the context of global ETEC and *E. coli* lineages, and linked the genomic diversity to traditional subtyping results based on serotyping and MLST-ST. In addition, we characterized the virulence factors, AMR mutation/gene and phenotype profiles of Shenzhen isolates. To our knowledge, this is the first study on the genomic epidemiology and AMR profile of ETEC infections in indigenous adults.

Shenzhen ETEC isolates showed a remarkable diversity, which can be attributed to four *E. coli* phylogroups, with the majority falling into phylogroup A (71%). The phylogroup distribution is different from that of global (von Mentzer et al., 2014) and Bangladeshi (Sahl et al., 2017) isolates [high fraction (>40%) of B1 isolates], and is similar to that of Chilean isolates (phylogroup A: 81%) (Rasko et al., 2019). More specifically, three major lineages, L11 (25%), L2/4 (21%), and L4 (15%), persisted in Shenzhen throughout the 6-year sampling period, together accounting for 60% cases. L11 can be detected in children aged <5 years and adult travelers from multiple countries, but at a low fraction (4%) in the global dataset (von Mentzer et al., 2014). If taken serotype and MLST-ST into consideration, L11 (O159:H34, ST218) isolates was only identified in local diarrheal cases of Korea (Chung et al., 2019) and China (Chen Y. et al., 2014; Pan et al., 2015; Li et al., 2017), and the virulence factor profiles (STh+, without known CS) of isolates from two countries were identical, indicating the possibility of recent transmission. L2/4 was a previously undefined lineage, which located between L2 and L4 in the phylogenic tree. However, it had a different virulence factor profile (STh: 21/22, STp: 1/22, CS21: 22/22) from L2 (global dataset: STh + LT, CS2 + CS3 ± CS21) or L4 (global dataset: STh/LT, CS6 ± CS21/CS6 + CS8/CS21). The serotype and MLST-ST of L2/4 was O6:H16 and ST48, respectively, and ETEC isolates with this subtyping combination were previously identified from pigs in Denmark (García et al., 2020) and from human cases in China (Chen Y. et al., 2014; Li et al., 2017). However, no CF was reported in Danish isolates whereas all Shenzhen isolates carried CS21. L4 lineage was also detected at low fraction (6%) in the global dataset (von Mentzer et al., 2014), and the subtyping combination of Shenzhen isolates, i.e., L4 (O25:H16, ST1491), was only identified in isolates from few travelers returning to the UK (Boxall et al., 2020) and from local diarrheal cases of China (Chen Y. et al., 2014; Pan et al., 2015; Li et al., 2017). Taken together, these results revealed that the major circulating ETEC lineages in Shenzhen, as well as in several other developed regions of China were distinctive, which are not the commonly detected global or endemic lineages.

In addition to subtyping, the virulence factor profile of Shenzhen and other Chinese ETEC isolates was also distinctive. ST-positive ETEC has been dominant (76%) in Shenzhen throughout the sampling period and 80% circulating lineages were ST-positive (53%) or contained ST-positive isolates (27%), whereas the fractions of LT-positive and ST + LT-positive isolates and lineages were lower. CS21 was the most prevalent CF (48%) in Shenzhen isolates, followed by CS6 (34%). Similar virulence factor profile was also observed in ETEC isolates from diarrheal cases in Shanghai, China (ST: 74%, CS21: 63%, CS6: 41%) (Pan et al., 2015). In contrast, a systematic review showed that globally, ST-positive, LT-positive, and ST + LT-positive ETEC accounted for 50, 25, and 27% non-travel human infections, respectively, and the most prevalent CFs were CFA/I, followed by CS21 and CS6 (Isidean et al., 2011). There was a considerable variability of virulence factor profiles across regions and populations. High ST-prevalence was only observed among travelers in East Asia/Pacific (75%) and non-travelers in Europe/Central Asia (77%), however, the most prevalent CF in East Asia/Pacific was CFA/I and CS21 was rarely detected (CF-prevalence in Europe/Central Asia is unavailable) (Isidean et al., 2011). Interestingly, CS21 was most prevalent CFs in ETEC-endemic regions including Latin America/Caribbean and the Middle East/North Africa, and among global travelers with a frequency of ∼22% (Isidean et al., 2011). Notably, the most commonly detected CF globally, CFA/I, was not identified in Shenzhen isolates. Moreover, a recent Global Enteric Multicenter Study (GEMS) report (Vidal et al., 2019) showed that the prevalence of ST, LT, and ST + LT among ETEC isolates from children aged <5 years with moderate-to-severe diarrhea in Africa and Asia were 36, 32, and 32%, respectively; the prevalence from the matched controls were 21, 47, and 33%, respectively. The most commonly detected CFs were CFA/IV (CS6 alone or with CS4 or CS5), CS5 + CS6, and CFA/I. Among all the sampling sites of this study, ST-positive ETEC isolates were not dominant (<50%), and CS21 was rarely detected (<2%). More specifically, the most prevalent ST variants in Shenzhen isolates were STa3/4 and STa5, which were consistent with that in global isolates (Joffré et al., 2016). However, the most prevalent LT variant in Shenzhen isolates was LT17. By contrast, the most prevalent LT variant in global isolates was LT1 (41%), while LT17 was rarely detected (2.1%) (Joffré et al., 2015).

The unique ETEC epidemiology in Shenzhen and China, i.e., most infections were from indigenous adults, might be related to several reasons. First, the pathogenicity of China ETEC isolates may be higher in adults than in children, given the distinctive lineage and virulence factor profiles. This hypothesis was supported by the observation in Guatemala (Torres et al., 2015), where ST-positive ETEC infections were significantly more prevalent in adult travelers compared to indigenous children, suggesting higher pathogenicity of ST-positive ETEC in adults, whereas most Chinese ETEC isolates were ST-positive. Moreover, a recent study showed that a ST enterotoxin variant STa5 was associated with ETEC infections in adults, suggesting that specific type of ETEC may have higher pathogenicity in adults (Joffré et al., 2016). However, the prevalence of STa5 variant in Shenzhen isolates was only 15%, indicating the existence of other known/unknown virulence genes associated with ETEC infections in adults. We identified multiple accessory genes significantly enriched in Shenzhen isolates by pan-genome analysis including the known virulence genes STh and *etpA*, which provides candidate targets for further studies. Second, different characterization between children and adult populations, such as eating habits and immunity, may also be associated with the unique ETEC epidemiology. For example, a study showed that dietary calcium can improve human resistance to ETEC infection (Bovee-Oudenhoven et al., 2003), while children usually tend to consume more calcium than the adult population. Besides, it has been reported that breastfeeding may protect infants against severe ETEC infection (Clemens et al., 1997). Furthermore, eating out is a major risk factor of foodborne disease (Liu et al., 2018), and the frequency in the adult population is usually higher than that in children. In addition, the immunity provided by previous ETEC infection or vaccine may decrease over age, leading to more ETEC infections in adults.

Characterizing the antibiotic susceptibility profile of ETEC would be helpful to guide the clinical treatment. In this study, we investigated the AMR mutation/genes and phenotype of Shenzhen ETEC isolates by integrating WGS-based analysis and antimicrobial susceptibility testing. AMR mutation/gene profile was generally concordant with the phenotype testing results of 52 representative isolates, which revealed high resistance rate to NAL (71%), AMP (69%), and AMS (46%). In recent years, high resistance of ETEC to these commonly used agents was also reported in multiple other countries including Peru (Rivera et al., 2010), Bangladesh (Begum et al., 2016), South Korea (Oh et al., 2014), and in other cities of China (Chen Y. et al., 2014; Pan et al., 2015). Due to the increased AMR, new antimicrobial agents such as azithromycin have been used as the first-line agent for ETEC infection treatment. Azithromycin is a broad-spectrum macrolide antimicrobial agent against several bacterial species, and is very effective for *Enterobacteriaceae* infection treatment (Gomes et al., 2019). However, azithromycin-resistant ETEC isolates from diarrheal patients have recently been reported in several countries at a moderate frequency (10–30%) (Begum et al., 2016; Guiral et al., 2019), and in Shanghai, China at a very high frequency (87%) (Xiang et al., 2020). Macrolides inactivation mediated by macrolide-resistant phosphotransferase *mph(A)* gene was the most common mechanism for the azithromycin resistance (Gomes et al., 2019; Xiang et al., 2020). We found that 38% representative Shenzhen ETEC isolates were resistant to azithromycin, and most of these isolates carried *mph(A)* gene. Moreover, we showed that the prevalence of ESBL-producing ETEC isolates from outpatients was 31% in Shenzhen, China, which is similar to that of diarrheal patients in Nepal post-2013 (30%) (Margulieux et al., 2018) and travel cases to Southeast Asia/India (43%) (Guiral et al., 2019). In addition, nearly all the isolates of a major circulating lineage L2/4 carried *gyrA* p.S83L mutation, *bla*_*TEM*–1*B*_ and *mph(A)* genes, and AMR phenotype testing showed that all the representative L2/4 isolates were resistant to AMP, AMS, and AZI and most (11/12) of them were resistant to NAL, suggesting that L2/4 was a MDR lineage. The identification of azithromycin-resistant, ESBL-producing ETEC and a major MDR lineage L2/4 in Shenzhen highlighted the importance of ongoing AMR surveillance.

In summary, during routine foodborne disease surveillance, we found that the epidemiology of ETEC infections in Shenzhen, China is distinctive, with most infections occurring in indigenous adults. By integrating WGS and antimicrobial susceptibility testing, we characterized the temporal dynamics of population structure and virulence factors, and the AMR mutation/gene and phenotype profile of Shenzhen ETEC isolates in 6 years. Shenzhen ETEC isolates showed a remarkable high diversity, which belonged to four *E. coli* phylogroups and 15 ETEC lineages, and the major virulence factors were enterotoxin ST and CF CS21 and CS6. Intriguingly, the major circulating lineages in Shenzhen and their virulence factor profiles were distinctive, which are different from the commonly detected global or endemic ETEC lineages. Furthermore, we showed that AMR mutation/gene profiles of genomes were concordant with the phenotype testing results, and revealed that Shenzhen isolates had high resistance rates to several commonly used antimicrobial agents and identified a MDR lineage. To our knowledge, our study provides novel insight into the genomic epidemiology and antimicrobial susceptibility profile of ETEC infections in indigenous adults for the first time, which will not only enhance our comprehensive understanding on ETEC epidemiology, but also have implications for the development of vaccine and future surveillance and prevention of ETEC infections.

## Data Availability Statement

The datasets presented in this study can be found in online repositories. The names of the repository/repositories and accession number(s) can be found in the article/Supplementary Material.

## Author Contributions

CY, YC, CW, and QH conceived and designed the study. CY, YL, LZ, MJ, LX, ML, YS, LW, YJ, SW, RC, and XS performed the experiments and data analysis. CY and LZ wrote the original draft. CY, XZ, YL, MJ, YC, CW, and QH reviewed and revised the manuscript. All authors have read and agreed to the published version of the manuscript.

## Conflict of Interest

The authors declare that the research was conducted in the absence of any commercial or financial relationships that could be construed as a potential conflict of interest.

## Publisher’s Note

All claims expressed in this article are solely those of the authors and do not necessarily represent those of their affiliated organizations, or those of the publisher, the editors and the reviewers. Any product that may be evaluated in this article, or claim that may be made by its manufacturer, is not guaranteed or endorsed by the publisher.

## Footnotes

1
https://github.com/phac-nml/ecoli_serotyping

2
https://github.com/tseemann/mlst

3
https://github.com/tseemann/abricate

4
https://github.com/avonm/ETEC_vir_db/

5
https://github.com/avonm/ETEC_toxin_variants_db

6
https://github.com/tseemann/snippy
