# Supplementary figures and images for: Genomic Epidemiology and Antimicrobial Susceptibility Profile of Enterotoxigenic Escherichia coli From Outpatients With Diarrhea in Shenzhen, China, 2015–2020
Source: Front Microbiol. 2021 Oct 28;12:732068. doi: 10.3389/fmicb.2021.732068 (PMC8581654; doi:10.3389/fmicb.2021.732068)

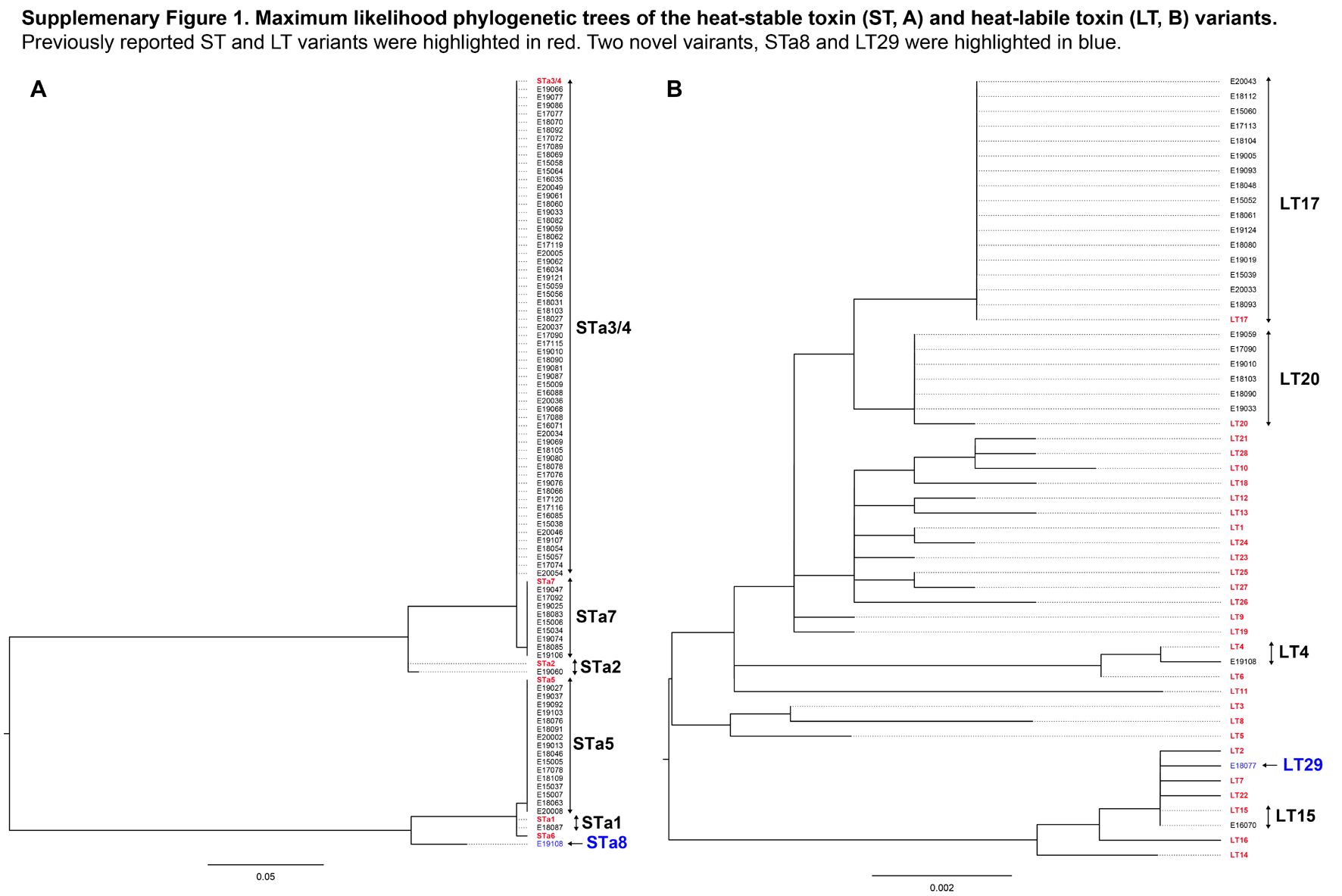

Supplement: Supplementary file 1 [file Image_1.tif]
